# Supplementary material for: Modeling Drug Resistance Emergence and Transmission in HIV-1 in the UK
Source: Viruses. 2023 May 25;15(6):1244. doi: 10.3390/v15061244 (PMC10304371; doi:10.3390/v15061244)
Supplement: Supplementary file 1 [file viruses-15-01244-s001.zip › viruses-2377830-supplementary.pdf]

Supplementary Materials: Modelling drug resistance emergence and transmission in HIV-1 in the UK

Anna Zhukova [1]*, David Dunn [2], Olivier Gascuel [3] *, on behalf of the UK HIV Drug Resistance Database & the Collaborative HIV, Anti-HIV Drug Resistance Network

1. Resistance statistics over time by DRM

1.1. Subtype B

Table S1. DRMs with prevalence > 0.5% found in position PR:I54 in B data set, and the evolution of their presence over time.

date	total samples	DRM	resistant cases (% of all)	resistant cases: treatment-experienced (% of resistant)	resistant cases: treatment-naive (% of resistant)	TDR cases (% of resistant)	TDR cluster num.	TDR cluster sizes	ADR cases (% of resistant)	loss cases (% of resistant)
14-03-16	39159	V	243 (0.6%)	182 (74.9%)	33 (13.6%)	62.52 (25.7%)	43	1-6	191.48 (78.8%)	11 (4.5%)
17-12-11	28011	V	231 (0.8%)	177 (76.6%)	27 (11.7%)	55.02 (23.8%)	39	1-6	184.98 (80.1%)	9 (3.9%)
17-12-06	13280	V	190 (1.4%)	152 (80.0%)	20 (10.5%)	36.12 (19.0%)	29	1-3	158.88 (83.6%)	5 (2.6%)
17-12-01	3195	V	82 (2.6%)	74 (90.2%)	4 (4.9%)	6.00 (7.3%)	6	1-1	76.00 (92.7%)	
14-11-96	7	V								

Table S2. DRMs with prevalence > 0.5% found in position PR:L33 in B data set, and the evolution of their presence over time.

date	total samples	DRM	resistant cases (% of all)	resistant cases: treatment-experienced (% of resistant)	resistant cases: treatment-naive (% of resistant)	TDR cases (% of resistant)	TDR cluster num.	TDR cluster sizes	ADR cases (% of resistant)	loss cases (% of resistant)
14-03-16	39159	F	230 (0.6%)	117 (50.9%)	92 (40.0%)	126.12 (54.8%)	70	1-10	114.88 (49.9%)	11 (4.8%)
17-12-11	28011	F	191 (0.7%)	104 (54.5%)	71 (37.2%)	95.75 (50.1%)	53.5	1-9	102.25 (53.5%)	7 (3.7%)
17-12-06	13280	F	111 (0.8%)	72 (64.9%)	28 (25.2%)	38.50 (34.7%)	27.5	1-5	76.50 (68.9%)	4 (3.6%)
17-12-01	3195	F	26 (0.8%)	22 (84.6%)	2 (7.7%)	3.00 (11.5%)	3	1-1	23.00 (88.5%)	
14-11-96	7	F								

Table S3. DRMs with prevalence > 0.5% found in position PR:L90 in B data set, and the evolution of their presence over time.

date	total samples	DRM	resistant cases (% of all)	resistant cases: treatment-experienced (% of resistant)	resistant cases: treatment-naive (% of resistant)	TDR cases (% of resistant)	TDR cluster num.	TDR cluster sizes	ADR cases (% of resistant)	loss cases (% of resistant)
14-03-16	39159	M	849 (2.2%)	480 (56.5%)	289 (34.0%)	460.77 (54.3%)	128	1-114	450.23 (53.0%)	62 (7.3%)
17-12-11	28011	M	709 (2.5%)	449 (63.3%)	196 (27.6%)	318.52 (44.9%)	109	1-71	435.48 (61.4%)	45 (6.3%)
17-12-06	13280	M	513 (3.9%)	390 (76.0%)	88 (17.2%)	137.62 (26.8%)	81	1-25	386.38 (75.3%)	11 (2.1%)
17-12-01	3195	M	206 (6.4%)	178 (86.4%)	16 (7.8%)	25.00 (12.1%)	20	1-5	182.00 (88.3%)	1 (0.5%)
14-11-96	7	M								

Table S4. DRMs with prevalence > 0.5% found in position PR:M46 in B data set, and the evolution of their presence over time.

**date**	**total** samples	**DRM**	**resistant cases** (% of all)	treatment-experienced (% of resistant)	naive (% of resistant)	**TDR** cases (% of resistant)	cluster num.	cluster sizes	**ADR** cases (% of resistant)	**loss** cases (% of resistant)
14-03-16	39159	I	378 (1.0%)	246 (65.1%)	97 (25.7%)	140.39 (37.1%)	108.5	1-6	250.61 (66.3%)	13 (3.4%)
17-12-11	28011	I	324 (1.2%)	232 (71.6%)	65 (20.1%)	95.52 (29.5%)	75.5	1-6	235.48 (72.7%)	7 (2.2%)
17-12-06	13280	I	238 (1.8%)	191 (80.3%)	30 (12.6%)	48.12 (20.2%)	41	1-4	194.88 (81.9%)	5 (2.1%)
17-12-01	3195	I	101 (3.2%)	91 (90.1%)	6 (5.9%)	8.00 (7.9%)	8	1-1	93.00 (92.1%)	
14-11-96	7	I	1 (14.3%)	1 (100.0%)					1.00 (100.0%)	

Table S5. DRMs with prevalence > 0.5% found in position PR:V82 in B data set, and the evolution of their presence over time.

**date**	**total** samples	**DRM**	**resistant cases** (% of all)	treatment-experienced (% of resistant)	naive (% of resistant)	**TDR** cases (% of resistant)	cluster num.	cluster sizes	**ADR** cases (% of resistant)	**loss** cases (% of resistant)
14-03-16	39159	A	295 (0.8%)	216 (73.2%)	51 (17.3%)	88.02 (29.8%)	53	1-11	218.98 (74.2%)	12 (4.1%)
17-12-11	28011	A	276 (1.0%)	210 (76.1%)	42 (15.2%)	74.52 (27.0%)	48	1-10	212.48 (77.0%)	11 (4.0%)
17-12-06	13280	A	220 (1.7%)	179 (81.4%)	25 (11.4%)	42.12 (19.1%)	33	1-3	183.88 (83.6%)	6 (2.7%)
17-12-01	3195	A	99 (3.1%)	92 (92.9%)	3 (3.0%)	6.00 (6.1%)	6	1-2	93.00 (93.9%)	
14-11-96	7	A								

Table S6. DRMs with prevalence > 0.5% found in position RT:A62 in B data set, and the evolution of their presence over time.

**date**	**total** samples	**DRM**	**resistant cases** (% of all)	treatment-experienced (% of resistant)	naive (% of resistant)	**TDR** cases (% of resistant)	cluster num.	cluster sizes	**ADR** cases (% of resistant)	**loss** cases (% of resistant)
14-03-16	39159	V	251 (0.6%)	147 (58.6%)	81 (32.3%)	114.50 (45.6%)	58.5	1-27	147.50 (58.8%)	11 (4.4%)
17-12-11	28011	V	208 (0.7%)	134 (64.4%)	57 (27.4%)	80.00 (38.5%)	42	1-16	134.00 (64.4%)	6 (2.9%)
17-12-06	13280	V	136 (1.0%)	109 (80.1%)	17 (12.5%)	28.50 (21.0%)	18.5	1-8	108.50 (79.8%)	1 (0.7%)
17-12-01	3195	V	55 (1.7%)	45 (81.8%)	6 (10.9%)	11.00 (20.0%)	8	1-4	44.00 (80.0%)	
14-11-96	7	V								

1.2. Subtype C

Table S7. DRMs with prevalence > 0.5% found in position RT:D67 in B data set, and the evolution of their presence over time.

date	total	DRM	resistant cases			TDR			ADR	loss
	samples		(% of all)	treatment-experienced (% of resistant)	treatment-naive (% of resistant)	cases (% of resistant)	cluster num.	cluster sizes	cases (% of resistant)	cases (% of resistant)
14-03-16	39159	N	1035 (2.6%)	806 (77.9%)	150 (14.5%)	273.00 (26.4%)	170.5	1-21	794.00 (76.7%)	32 (3.1%)
17-12-11	28011	N	965 (3.4%)	772 (80.0%)	126 (13.1%)	222.25 (23.0%)	144	1-20	766.75 (79.5%)	24 (2.5%)
17-12-06	13280	N	801 (6.0%)	670 (83.6%)	76 (9.5%)	139.50 (17.4%)	105.5	1-10	676.50 (84.5%)	15 (1.9%)
17-12-01	3195	N	364 (11.4%)	329 (90.4%)	20 (5.5%)	32.50 (8.9%)	31.5	1-2	332.50 (91.3%)	1 (0.3%)
14-11-96	7	N	4 (57.1%)	4 (100.0%)					4.00 (100.0%)	

Table S8. DRMs with prevalence > 0.5% found in position RT:E138 in B data set, and the evolution of their presence over time.

date	total	DRM	resistant cases			TDR			ADR	loss
	samples		(% of all)	treatment-experienced (% of resistant)	treatment-naive (% of resistant)	cases (% of resistant)	cluster num.	cluster sizes	cases (% of resistant)	cases (% of resistant)
14-03-16	39159	A	862 (2.2%)	163 (18.9%)	637 (73.9%)	760.25 (88.2%)	305.5	1-158	155.75 (18.1%)	54 (6.3%)
17-12-11	28011	A	582 (2.1%)	124 (21.3%)	428 (73.5%)	485.75 (83.5%)	226	1-89	122.25 (21.0%)	26 (4.5%)
17-12-06	13280	A	239 (1.8%)	87 (36.4%)	142 (59.4%)	164.75 (68.9%)	107.5	1-14	84.25 (35.3%)	10 (4.2%)
17-12-01	3195	A	61 (1.9%)	31 (50.8%)	29 (47.5%)	33.50 (54.9%)	28.5	1-4	29.50 (48.4%)	2 (3.3%)
14-11-96	7	A								

Table S9. DRMs with prevalence > 0.5% found in position RT:E44 in B data set, and the evolution of their presence over time.

date	total	DRM	resistant cases			TDR			ADR	loss
	samples		(% of all)	treatment-experienced (% of resistant)	treatment-naive (% of resistant)	cases (% of resistant)	cluster num.	cluster sizes	cases (% of resistant)	cases (% of resistant)
14-03-16	39159	D	294 (0.8%)	180 (61.2%)	93 (31.6%)	129.62 (44.1%)	77	1-29	183.38 (62.4%)	19 (6.5%)
17-12-11	28011	D	249 (0.9%)	170 (68.3%)	60 (24.1%)	87.50 (35.1%)	58	1-9	176.50 (70.9%)	15 (6.0%)
17-12-06	13280	D	197 (1.5%)	149 (75.6%)	31 (15.7%)	49.75 (25.3%)	37.5	1-3	156.25 (79.3%)	9 (4.6%)
17-12-01	3195	D	79 (2.5%)	66 (83.5%)	7 (8.9%)	11.00 (13.9%)	10	1-2	70.00 (88.6%)	2 (2.5%)
14-11-96	7	D	1 (14.3%)	1 (100.0%)					1.00 (100.0%)	

Table S10. DRMs with prevalence > 0.5% found in position RT:G190 in B data set, and the evolution of their presence over time.

date	total	DRM	resistant cases			TDR			ADR	loss
	samples		(% of all)	treatment-experienced (% of resistant)	treatment-naive (% of resistant)	cases (% of resistant)	cluster num.	cluster sizes	cases (% of resistant)	cases (% of resistant)
14-03-16	39159	A	447 (1.1%)	342 (76.5%)	68 (15.2%)	117.25 (26.2%)	97	1-6	350.75 (78.5%)	21 (4.7%)
17-12-11	28011	A	395 (1.4%)	312 (79.0%)	57 (14.4%)	88.00 (22.3%)	77	1-4	319.00 (80.8%)	12 (3.0%)
17-12-06	13280	A	341 (2.6%)	280 (82.1%)	38 (11.1%)	60.00 (17.6%)	58	1-2	287.00 (84.2%)	6 (1.8%)
17-12-01	3195	A	99 (3.1%)	89 (89.9%)	6 (6.1%)	8.00 (8.1%)	8	1-2	91.00 (91.9%)	
14-11-96	7	A								

Table S11. DRMs with prevalence > 0.5% found in position RT:H221 in B data set, and the evolution of their presence over time.

date	total samples	DRM	resistant cases (% of all)	resistant cases: treatment-experienced (% of resistant)	resistant cases: treatment-naive (% of resistant)	TDR cases (% of resistant)	TDR cluster num.	TDR cluster sizes	ADR cases (% of resistant)	loss cases (% of resistant)
14-03-16	39159	Y	475 (1.2%)	269 (56.6%)	162 (34.1%)	220.00 (46.3%)	87	1-64	267.00 (56.2%)	12 (2.5%)
17-12-11	28011	Y	403 (1.4%)	240 (59.6%)	132 (32.8%)	169.50 (42.1%)	65.5	1-49	242.50 (60.2%)	9 (2.2%)
17-12-06	13280	Y	251 (1.9%)	185 (73.7%)	48 (19.1%)	66.50 (26.5%)	43.5	1-9	188.50 (75.1%)	4 (1.6%)
17-12-01	3195	Y	61 (1.9%)	47 (77.0%)	11 (18.0%)	12.50 (20.5%)	12.5	1-1	48.50 (79.5%)	
14-11-96	7	Y	1 (14.3%)	1 (100.0%)					1.00 (100.0%)	

Table S12. DRMs with prevalence > 0.5% found in position RT:K101 in B data set, and the evolution of their presence over time.

date	total samples	DRM	resistant cases (% of all)	resistant cases: treatment-experienced (% of resistant)	resistant cases: treatment-naive (% of resistant)	TDR cases (% of resistant)	TDR cluster num.	TDR cluster sizes	ADR cases (% of resistant)	loss cases (% of resistant)
14-03-16	39159	E	276 (0.7%)	189 (68.5%)	64 (23.2%)	94.50 (34.2%)	71.5	1-9	190.50 (69.0%)	9 (3.3%)
17-12-11	28011	E	223 (0.8%)	161 (72.2%)	45 (20.2%)	61.00 (27.4%)	51.5	1-3	166.00 (74.4%)	4 (1.8%)
17-12-06	13280	E	176 (1.3%)	141 (80.1%)	24 (13.6%)	35.50 (20.2%)	31.5	1-2	143.50 (81.5%)	3 (1.7%)
17-12-01	3195	E	44 (1.4%)	40 (90.9%)	2 (4.5%)	3.00 (6.8%)	3	1-1	41.00 (93.2%)	
14-11-96	7	E								

Table S13. DRMs with prevalence > 0.5% found in position RT:K103 in B data set, and the evolution of their presence over time.

date	total samples	DRM	resistant cases (% of all)	resistant cases: treatment-experienced (% of resistant)	resistant cases: treatment-naive (% of resistant)	TDR cases (% of resistant)	TDR cluster num.	TDR cluster sizes	ADR cases (% of resistant)	loss cases (% of resistant)
14-03-16	39159	N	2025 (5.2%)	1104 (54.5%)	745 (36.8%)	1071.51 (52.9%)	516.5	1-78	1088.49 (53.8%)	135 (6.7%)
17-12-11	28011	N	1584 (5.7%)	925 (58.4%)	540 (34.1%)	722.25 (45.6%)	377.5	1-57	933.75 (58.9%)	72 (4.5%)
17-12-06	13280	N	1042 (7.8%)	731 (70.2%)	228 (21.9%)	323.00 (31.0%)	219	1-16	745.00 (71.5%)	26 (2.5%)
17-12-01	3195	N	269 (8.4%)	230 (85.5%)	24 (8.9%)	33.50 (12.5%)	33.5	1-2	235.50 (87.5%)	
14-11-96	7	N								

Table S14. DRMs with prevalence > 0.5% found in position RT:K219 in B data set, and the evolution of their presence over time.

**date**	**total** samples	**DRM**	**resistant cases** (% of all)	treatment-experienced (% of resistant)	naive (% of resistant)	**TDR** cases (% of resistant)	cluster num.	cluster sizes	**ADR** cases (% of resistant)	**loss** cases (% of resistant)
14-03-16	39159	E	262 (0.7%)	192 (73.3%)	43 (16.4%)	74.75 (28.5%)	51.5	1-9	192.25 (73.4%)	5 (1.9%)
		N	238 (0.6%)	92 (38.7%)	127 (53.4%)	161.00 (67.6%)	23	1-113	81.00 (34.0%)	4 (1.7%)
		Q	563 (1.4%)	322 (57.2%)	194 (34.5%)	307.25 (54.6%)	99	1-92	303.75 (54.0%)	48 (8.5%)
17-12-11	28011	E	227 (0.8%)	181 (79.7%)	31 (13.7%)	50.50 (22.2%)	34	1-9	180.50 (79.5%)	4 (1.8%)
		N	194 (0.7%)	83 (42.8%)	101 (52.1%)	119.50 (61.6%)	17.5	1-86	76.50 (39.4%)	2 (1.0%)
		Q	497 (1.8%)	299 (60.2%)	164 (33.0%)	245.75 (49.4%)	80.5	1-78	289.25 (58.2%)	38 (7.6%)
17-12-06	13280	E	169 (1.3%)	145 (85.8%)	11 (6.5%)	25.50 (15.1%)	21.5	1-2	147.50 (87.3%)	4 (2.4%)
		N	101 (0.8%)	66 (65.3%)	32 (31.7%)	40.50 (40.1%)	13.5	1-17	62.50 (61.9%)	2 (2.0%)
		Q	365 (2.7%)	256 (70.1%)	86 (23.6%)	129.25 (35.4%)	57	1-32	256.75 (70.3%)	21 (5.8%)
17-12-01	3195	E	74 (2.3%)	67 (90.5%)	5 (6.8%)	7.00 (9.5%)	7	1-2	67.00 (90.5%)	
		N	26 (0.8%)	20 (76.9%)	5 (19.2%)	5.50 (21.2%)	3.5	1-3	20.50 (78.8%)	
		Q	147 (4.6%)	127 (86.4%)	15 (10.2%)	21.50 (14.6%)	17.5	1-2	128.50 (87.4%)	3 (2.0%)
14-11-96	7	E								
		N	1 (14.3%)	1 (100.0%)					1.00 (100.0%)	
		Q	1 (14.3%)	1 (100.0%)					1.00 (100.0%)	

Table S15. DRMs with prevalence > 0.5% found in position RT:K65 in B data set, and the evolution of their presence over time.

**date**	**total** samples	**DRM**	**resistant cases** (% of all)	treatment-experienced (% of resistant)	naive (% of resistant)	**TDR** cases (% of resistant)	cluster num.	cluster sizes	**ADR** cases (% of resistant)	**loss** cases (% of resistant)
14-03-16	39159	R	225 (0.6%)	170 (75.6%)	19 (8.4%)	50.88 (22.6%)	42	1-2	187.12 (83.2%)	13 (5.8%)
17-12-11	28011	R	189 (0.7%)	146 (77.2%)	15 (7.9%)	37.62 (19.9%)	33	1-2	159.38 (84.3%)	8 (4.2%)
17-12-06	13280	R	143 (1.1%)	114 (79.7%)	8 (5.6%)	23.12 (16.2%)	20.5	1-2	123.88 (86.6%)	4 (2.8%)
17-12-01	3195	R	19 (0.6%)	18 (94.7%)		0.50 (2.6%)	0.5	1-1	18.50 (97.4%)	
14-11-96	7	R								

Table S16. DRMs with prevalence > 0.5% found in position RT:K70 in B data set, and the evolution of their presence over time.

**date**	**total** samples	**DRM**	**resistant cases** (% of all)	treatment-experienced (% of resistant)	naive (% of resistant)	**TDR** cases (% of resistant)	cluster num.	cluster sizes	**ADR** cases (% of resistant)	**loss** cases (% of resistant)
14-03-16	39159	R	711 (1.8%)	610 (85.8%)	54 (7.6%)	143.75 (20.2%)	98.5	1-7	615.25 (86.5%)	48 (6.8%)
17-12-11	28011	R	681 (2.4%)	596 (87.5%)	46 (6.8%)	120.50 (17.7%)	84	1-5	602.50 (88.5%)	42 (6.2%)
17-12-06	13280	R	604 (4.5%)	534 (88.4%)	36 (6.0%)	91.00 (15.1%)	67	1-4	541.00 (89.6%)	28 (4.6%)
17-12-01	3195	R	294 (9.2%)	269 (91.5%)	18 (6.1%)	27.50 (9.4%)	25.5	1-2	269.50 (91.7%)	3 (1.0%)
14-11-96	7	R	2 (28.6%)	2 (100.0%)					2.00 (100.0%)	

Table S17. DRMs with prevalence > 0.5% found in position RT:L210 in B data set, and the evolution of their presence over time.

date	total samples	DRM	resistant cases (% of all)	resistant cases treatment-experienced (% of resistant)	resistant cases treatment-naive (% of resistant)	TDR cases (% of resistant)	TDR cluster num.	TDR cluster sizes	ADR cases (% of resistant)	loss cases (% of resistant)
14-03-16	39159	W	705 (1.8%)	520 (73.8%)	140 (19.9%)	205.00 (29.1%)	147	1-9	524.00 (74.3%)	24 (3.4%)
17-12-11	28011	W	649 (2.3%)	501 (77.2%)	113 (17.4%)	161.75 (24.9%)	121	1-7	506.25 (78.0%)	19 (2.9%)
17-12-06	13280	W	556 (4.2%)	452 (81.3%)	70 (12.6%)	106.75 (19.2%)	81	1-7	460.25 (82.8%)	11 (2.0%)
17-12-01	3195	W	236 (7.4%)	203 (86.0%)	20 (8.5%)	26.50 (11.2%)	22.5	1-4	209.50 (88.8%)	
14-11-96	7	W	3 (42.9%)	3 (100.0%)					3.00 (100.0%)	

Table S18. DRMs with prevalence > 0.5% found in position RT:L74 in B data set, and the evolution of their presence over time.

date	total samples	DRM	resistant cases (% of all)	resistant cases treatment-experienced (% of resistant)	resistant cases treatment-naive (% of resistant)	TDR cases (% of resistant)	TDR cluster num.	TDR cluster sizes	ADR cases (% of resistant)	loss cases (% of resistant)
14-03-16	39159	V	242 (0.6%)	200 (82.6%)	17 (7.0%)	38.25 (15.8%)	36	1-3	207.75 (85.8%)	4 (1.7%)
17-12-11	28011	V	216 (0.8%)	182 (84.3%)	15 (6.9%)	30.50 (14.1%)	29.5	1-3	189.50 (87.7%)	4 (1.9%)
17-12-06	13280	V	172 (1.3%)	147 (85.5%)	11 (6.4%)	21.50 (12.5%)	21.5	1-2	152.50 (88.7%)	2 (1.2%)
17-12-01	3195	V	54 (1.7%)	48 (88.9%)	2 (3.7%)	4.00 (7.4%)	4	1-1	50.00 (92.6%)	
14-11-96	7	V								

Table S19. DRMs with prevalence > 0.5% found in position RT:M184 in B data set, and the evolution of their presence over time.

date	total samples	DRM	resistant cases (% of all)	resistant cases treatment-experienced (% of resistant)	resistant cases treatment-naive (% of resistant)	TDR cases (% of resistant)	TDR cluster num.	TDR cluster sizes	ADR cases (% of resistant)	loss cases (% of resistant)
14-03-16	39159	V	1899 (4.8%)	1642 (86.5%)	110 (5.8%)	343.62 (18.1%)	278.5	1-4	1667.38 (87.8%)	112 (5.9%)
17-12-11	28011	V	1703 (6.1%)	1493 (87.7%)	94 (5.5%)	266.62 (15.7%)	213.5	1-4	1517.38 (89.1%)	81 (4.8%)
17-12-06	13280	V	1428 (10.8%)	1259 (88.2%)	74 (5.2%)	190.56 (13.3%)	156.5	1-3	1284.44 (89.9%)	47 (3.3%)
17-12-01	3195	V	603 (18.9%)	551 (91.4%)	22 (3.6%)	50.56 (8.4%)	45	1-3	560.44 (92.9%)	8 (1.3%)
14-11-96	7	V	2 (28.6%)	2 (100.0%)					2.00 (100.0%)	

Table S20. DRMs with prevalence > 0.5% found in position RT:M41 in B data set, and the evolution of their presence over time.

date	total samples	DRM	resistant cases (% of all)	resistant cases treatment-experienced (% of resistant)	resistant cases treatment-naive (% of resistant)	TDR cases (% of resistant)	TDR cluster num.	TDR cluster sizes	ADR cases (% of resistant)	loss cases (% of resistant)
14-03-16	39159	L	1513 (3.9%)	982 (64.9%)	428 (28.3%)	618.50 (40.9%)	305.5	1-55	968.50 (64.0%)	74 (4.9%)
17-12-11	28011	L	1389 (5.0%)	938 (67.5%)	367 (26.4%)	507.50 (36.5%)	266.5	1-38	935.50 (67.4%)	54 (3.9%)
17-12-06	13280	L	1099 (8.3%)	826 (75.2%)	208 (18.9%)	294.00 (26.8%)	188	1-16	830.00 (75.5%)	25 (2.3%)
17-12-01	3195	L	459 (14.4%)	386 (84.1%)	52 (11.3%)	68.75 (15.0%)	56	1-6	392.25 (85.5%)	2 (0.4%)
14-11-96	7	L	2 (28.6%)	2 (100.0%)					2.00 (100.0%)	

Table S21. DRMs with prevalence > 0.5% found in position RT:S68 in B data set, and the evolution of their presence over time.

date	total samples	DRM	resistant cases (% of all)	treatment-experienced (% of resistant)	treatment-naive (% of resistant)	TDR cases (% of resistant)	cluster num.	cluster sizes	ADR cases (% of resistant)	loss cases (% of resistant)
14-03-16	39159	G	3178 (8.1%)	436 (13.7%)	2482 (78.1%)	2922.56 (92.0%)	612.5	1-759	316.44 (10.0%)	61 (1.9%)
17-12-11	28011	G	2055 (7.3%)	318 (15.5%)	1601 (77.9%)	1845.00 (89.8%)	468.5	1-486	251.00 (12.2%)	41 (2.0%)
17-12-06	13280	G	868 (6.5%)	204 (23.5%)	610 (70.3%)	721.00 (83.1%)	234.5	1-174	166.00 (19.1%)	19 (2.2%)
17-12-01	3195	G	148 (4.6%)	63 (42.6%)	78 (52.7%)	98.50 (66.6%)	60	1-22	54.50 (36.8%)	5 (3.4%)
14-11-96	7	G								

Table S22. DRMs with prevalence > 0.5% found in position RT:T215 in B data set, and the evolution of their presence over time.

date	total samples	DRM	resistant cases (% of all)	treatment-experienced (% of resistant)	treatment-naive (% of resistant)	TDR cases (% of resistant)	cluster num.	cluster sizes	ADR cases (% of resistant)	loss cases (% of resistant)
14-03-16	39159	D	462 (1.2%)	86 (18.6%)	334 (72.3%)	459.25 (99.4%)	103	1-99	71.75 (15.5%)	69 (14.9%)
		F	257 (0.7%)	215 (83.7%)	19 (7.4%)	41.25 (16.1%)	37	1-4	222.75 (86.7%)	7 (2.7%)
		S	378 (1.0%)	59 (15.6%)	293 (77.5%)	364.25 (96.4%)	115	1-45	51.75 (13.7%)	38 (10.1%)
		Y	883 (2.3%)	790 (89.5%)	37 (4.2%)	119.50 (13.5%)	102.5	1-5	785.50 (89.0%)	22 (2.5%)
17-12-11	28011	D	367 (1.3%)	68 (18.5%)	272 (74.1%)	366.50 (99.9%)	91	1-68	62.50 (17.0%)	62 (16.9%)
		F	246 (0.9%)	209 (85.0%)	17 (6.9%)	36.50 (14.8%)	33.5	1-4	214.50 (87.2%)	5 (2.0%)
		S	282 (1.0%)	37 (13.1%)	228 (80.9%)	276.75 (98.1%)	90	1-30	38.25 (13.6%)	33 (11.7%)
		Y	858 (3.1%)	770 (89.7%)	36 (4.2%)	105.00 (12.2%)	93	1-5	768.00 (89.5%)	15 (1.7%)
17-12-06	13280	D	198 (1.5%)	44 (22.2%)	143 (72.2%)	187.00 (94.4%)	63	1-34	48.00 (24.2%)	37 (18.7%)
		F	221 (1.7%)	187 (84.6%)	16 (7.2%)	30.50 (13.8%)	29.5	1-3	193.50 (87.6%)	3 (1.4%)
		S	128 (1.0%)	24 (18.8%)	93 (72.7%)	125.50 (98.0%)	50.5	1-10	28.50 (22.3%)	26 (20.3%)
		Y	777 (5.9%)	696 (89.6%)	33 (4.2%)	82.00 (10.6%)	73	1-5	701.00 (90.2%)	6 (0.8%)
17-12-01	3195	D	47 (1.5%)	13 (27.7%)	31 (66.0%)	35.50 (75.5%)	21.5	1-4	14.50 (30.9%)	3 (6.4%)
		F	98 (3.1%)	91 (92.9%)	5 (5.1%)	8.00 (8.2%)	8	1-2	90.00 (91.8%)	
		S	23 (0.7%)	7 (30.4%)	14 (60.9%)	17.00 (73.9%)	9.5	1-3	9.00 (39.1%)	3 (13.0%)
		Y	362 (11.3%)	334 (92.3%)	11 (3.0%)	21.75 (6.0%)	21.5	1-2	341.25 (94.3%)	1 (0.3%)
14-11-96	7	D	1 (14.3%)	1 (100.0%)					1.00 (100.0%)	
		F	1 (14.3%)	1 (100.0%)					1.00 (100.0%)	
		S								
		Y	2 (28.6%)	2 (100.0%)					2.00 (100.0%)	

Table S23. DRMs with prevalence > 0.5% found in position RT:V106 in B data set, and the evolution of their presence over time.

date	total samples	DRM	resistant cases (% of all)	treatment-experienced (% of resistant)	treatment-naive (% of resistant)	TDR cases (% of resistant)	cluster num.	cluster sizes	ADR cases (% of resistant)	loss cases (% of resistant)
14-03-16	39159	I	1051 (2.7%)	217 (20.6%)	715 (68.0%)	956.16 (91.0%)	494.5	1-74	230.84 (22.0%)	136 (12.9%)
17-12-11	28011	I	717 (2.6%)	152 (21.2%)	494 (68.9%)	651.38 (90.8%)	340	1-57	164.62 (23.0%)	99 (13.8%)
17-12-06	13280	I	321 (2.4%)	94 (29.3%)	201 (62.6%)	262.75 (81.9%)	158	1-16	100.25 (31.2%)	42 (13.1%)
17-12-01	3195	I	64 (2.0%)	29 (45.3%)	35 (54.7%)	45.00 (70.3%)	31	1-3	29.00 (45.3%)	10 (15.6%)
14-11-96	7	I								

Table S24. DRMs with prevalence > 0.5% found in position RT:V108 in B data set, and the evolution of their presence over time.

**date**	**total** samples	**DRM**	**resistant cases** (% of all)	treatment-experienced (% of resistant)	naive (% of resistant)	**TDR** cases (% of resistant)	cluster num.	cluster sizes	**ADR** cases (% of resistant)	**loss** cases (% of resistant)
14-03-16	39159	I	429 (1.1%)	219 (51.0%)	167 (38.9%)	230.00 (53.6%)	166	1-8	232.00 (54.1%)	33 (7.7%)
17-12-11	28011	I	338 (1.2%)	186 (55.0%)	126 (37.3%)	161.25 (47.7%)	127.5	1-4	195.75 (57.9%)	19 (5.6%)
17-12-06	13280	I	224 (1.7%)	134 (59.8%)	73 (32.6%)	89.50 (40.0%)	76.5	1-4	139.50 (62.3%)	5 (2.2%)
17-12-01	3195	I	53 (1.7%)	36 (67.9%)	13 (24.5%)	15.00 (28.3%)	14	1-2	38.00 (71.7%)	
14-11-96	7	I								

Table S25. DRMs with prevalence > 0.5% found in position RT:V179 in B data set, and the evolution of their presence over time.

**date**	**total** samples	**DRM**	**resistant cases** (% of all)	treatment-experienced (% of resistant)	naive (% of resistant)	**TDR** cases (% of resistant)	cluster num.	cluster sizes	**ADR** cases (% of resistant)	**loss** cases (% of resistant)
14-03-16	39159	D	790 (2.0%)	151 (19.1%)	559 (70.8%)	694.12 (87.9%)	334.5	1-45	158.88 (20.1%)	63 (8.0%)
17-12-11	28011	D	539 (1.9%)	112 (20.8%)	371 (68.8%)	462.06 (85.7%)	239	1-38	122.94 (22.8%)	46 (8.5%)
17-12-06	13280	D	225 (1.7%)	76 (33.8%)	128 (56.9%)	165.50 (73.6%)	105	1-23	82.50 (36.7%)	23 (10.2%)
17-12-01	3195	D	41 (1.3%)	23 (56.1%)	16 (39.0%)	20.00 (48.8%)	16	1-3	23.00 (56.1%)	2 (4.9%)
14-11-96	7	D								

Table S26. DRMs with prevalence > 0.5% found in position RT:Y181 in B data set, and the evolution of their presence over time.

**date**	**total** samples	**DRM**	**resistant cases** (% of all)	treatment-experienced (% of resistant)	naive (% of resistant)	**TDR** cases (% of resistant)	cluster num.	cluster sizes	**ADR** cases (% of resistant)	**loss** cases (% of resistant)
14-03-16	39159	C	694 (1.8%)	495 (71.3%)	115 (16.6%)	208.00 (30.0%)	148	1-12	509.00 (73.3%)	23 (3.3%)
17-12-11	28011	C	600 (2.1%)	442 (73.7%)	96 (16.0%)	159.50 (26.6%)	110	1-12	457.50 (76.2%)	17 (2.8%)
17-12-06	13280	C	459 (3.5%)	353 (76.9%)	56 (12.2%)	96.50 (21.0%)	79.5	1-5	370.50 (80.7%)	8 (1.7%)
17-12-01	3195	C	126 (3.9%)	112 (88.9%)	7 (5.6%)	10.50 (8.3%)	10.5	1-1	115.50 (91.7%)	
14-11-96	7	C								

Table S27. DRMs with prevalence > 0.5% found in position PR:L90 in C data set, and the evolution of their presence over time.

**date**	**total** samples	**DRM**	**resistant cases** (% of all)	treatment-experienced (% of resistant)	naive (% of resistant)	**TDR** cases (% of resistant)	cluster num.	cluster sizes	**ADR** cases (% of resistant)	**loss** cases (% of resistant)
14-03-16	18809	M	108 (0.6%)	62 (57.4%)	22 (20.4%)	43.00 (39.8%)	35.5	1-4	67.00 (62.0%)	2 (1.9%)
17-12-11	14035	M	91 (0.6%)	57 (62.6%)	14 (15.4%)	31.25 (34.3%)	27	1-3	60.75 (66.8%)	1 (1.1%)
17-12-06	5374	M	68 (1.3%)	47 (69.1%)	6 (8.8%)	16.00 (23.5%)	15	1-3	52.00 (76.5%)	
17-12-01	338	M	11 (3.3%)	7 (63.6%)	1 (9.1%)	2.50 (22.7%)	2.5	1-1	8.50 (77.3%)	

Table S28. DRMs with prevalence > 0.5% found in position PR:Q58 in C data set, and the evolution of their presence over time.

date	total	DRM	resistant cases			TDR			ADR	loss
	samples		(% of all)	treatment-experienced (% of resistant)	naive (% of resistant)	cases (% of resistant)	cluster num.	cluster sizes	cases (% of resistant)	cases (% of resistant)
14-03-16	18809	E	153 (0.8%)	31 (20.3%)	97 (63.4%)	117.75 (77.0%)	81.5	1-12	37.25 (24.3%)	2 (1.3%)
17-12-11	14035	E	104 (0.7%)	24 (23.1%)	68 (65.4%)	79.00 (76.0%)	61	1-9	27.00 (26.0%)	2 (1.9%)
17-12-06	5374	E	34 (0.6%)	10 (29.4%)	20 (58.8%)	23.00 (67.6%)	21	1-2	12.00 (35.3%)	1 (2.9%)
17-12-01	338	E	1 (0.3%)			0.50 (50.0%)	0.5	1-1	0.50 (50.0%)	

Table S29. DRMs with prevalence > 0.5% found in position RT:A98 in C data set, and the evolution of their presence over time.

date	total	DRM	resistant cases			TDR			ADR	loss
	samples		(% of all)	treatment-experienced (% of resistant)	naive (% of resistant)	cases (% of resistant)	cluster num.	cluster sizes	cases (% of resistant)	cases (% of resistant)
14-03-16	18809	G	239 (1.3%)	115 (48.1%)	78 (32.6%)	112.12 (46.9%)	99	1-4	126.88 (53.1%)	
17-12-11	14035	G	187 (1.3%)	89 (47.6%)	65 (34.8%)	88.12 (47.1%)	77.5	1-4	98.88 (52.9%)	
17-12-06	5374	G	93 (1.7%)	55 (59.1%)	17 (18.3%)	32.31 (34.7%)	28.5	1-4	60.69 (65.3%)	
17-12-01	338	G	10 (3.0%)	7 (70.0%)	1 (10.0%)	2.25 (22.5%)	2	1-2	7.75 (77.5%)	

Table S30. DRMs with prevalence > 0.5% found in position RT:D67 in C data set, and the evolution of their presence over time.

date	total	DRM	resistant cases			TDR			ADR	loss
	samples		(% of all)	treatment-experienced (% of resistant)	naive (% of resistant)	cases (% of resistant)	cluster num.	cluster sizes	cases (% of resistant)	cases (% of resistant)
14-03-16	18809	N	289 (1.5%)	215 (74.4%)	25 (8.7%)	65.25 (22.6%)	56.5	1-4	228.75 (79.2%)	5 (1.7%)
17-12-11	14035	N	247 (1.8%)	187 (75.7%)	22 (8.9%)	49.00 (19.8%)	42.5	1-4	199.00 (80.6%)	1 (0.4%)
17-12-06	5374	N	166 (3.1%)	130 (78.3%)	11 (6.6%)	28.81 (17.4%)	26.5	1-4	138.19 (83.2%)	1 (0.6%)
17-12-01	338	N	23 (6.8%)	18 (78.3%)	1 (4.3%)	4.25 (18.5%)	4	1-2	18.75 (81.5%)	

Table S31. DRMs with prevalence > 0.5% found in position RT:E138 in C data set, and the evolution of their presence over time.

date	total	DRM	resistant cases			TDR			ADR	loss
	samples		(% of all)	treatment-experienced (% of resistant)	naive (% of resistant)	cases (% of resistant)	cluster num.	cluster sizes	cases (% of resistant)	cases (% of resistant)
14-03-16	18809	A	2176 (11.6%)	512 (23.5%)	1381 (63.5%)	2136.88 (98.2%)	415.5	1-1178	196.12 (9.0%)	157 (7.2%)
17-12-11	14035	A	1609 (11.5%)	331 (20.6%)	1132 (70.4%)	1609.25 (100.0%)	345	1-852	135.75 (8.4%)	136 (8.5%)
17-12-06	5374	A	617 (11.5%)	154 (25.0%)	417 (67.6%)	615.50 (99.8%)	150.5	1-322	65.50 (10.6%)	64 (10.4%)
17-12-01	338	A	28 (8.3%)	10 (35.7%)	12 (42.9%)	26.50 (94.6%)	5.5	1-18	6.50 (23.2%)	5 (17.9%)

Table S32. DRMs with prevalence > 0.5% found in position RT:G190 in C data set, and the evolution of their presence over time.

date	total samples	DRM	resistant cases (% of all)	resistant cases treatment-experienced (% of resistant)	resistant cases naive (% of resistant)	TDR cases (% of resistant)	TDR cluster num.	TDR cluster sizes	ADR cases (% of resistant)	loss cases (% of resistant)
14-03-16	18809	A	287 (1.5%)	213 (74.2%)	34 (11.8%)	71.25 (24.8%)	65.5	1-4	224.75 (78.3%)	9 (3.1%)
17-12-11	14035	A	233 (1.7%)	173 (74.2%)	27 (11.6%)	50.75 (21.8%)	47	1-4	184.25 (79.1%)	2 (0.9%)
17-12-06	5374	A	144 (2.7%)	111 (77.1%)	14 (9.7%)	27.50 (19.1%)	27	1-2	117.50 (81.6%)	1 (0.7%)
17-12-01	338	A	16 (4.7%)	13 (81.2%)	1 (6.2%)	3.00 (18.8%)	3	1-2	13.00 (81.2%)	

Table S33. DRMs with prevalence > 0.5% found in position RT:H221 in C data set, and the evolution of their presence over time.

date	total samples	DRM	resistant cases (% of all)	resistant cases treatment-experienced (% of resistant)	resistant cases naive (% of resistant)	TDR cases (% of resistant)	TDR cluster num.	TDR cluster sizes	ADR cases (% of resistant)	loss cases (% of resistant)
14-03-16	18809	Y	173 (0.9%)	123 (71.1%)	27 (15.6%)	45.75 (26.4%)	42.5	1-2	133.25 (77.0%)	6 (3.5%)
17-12-11	14035	Y	144 (1.0%)	102 (70.8%)	23 (16.0%)	36.25 (25.2%)	34	1-2	111.75 (77.6%)	4 (2.8%)
17-12-06	5374	Y	79 (1.5%)	57 (72.2%)	14 (17.7%)	19.75 (25.0%)	19.5	1-2	60.25 (76.3%)	1 (1.3%)
17-12-01	338	Y	5 (1.5%)	5 (100.0%)					5.00 (100.0%)	

Table S34. DRMs with prevalence > 0.5% found in position RT:K101 in C data set, and the evolution of their presence over time.

date	total samples	DRM	resistant cases (% of all)	resistant cases treatment-experienced (% of resistant)	resistant cases naive (% of resistant)	TDR cases (% of resistant)	TDR cluster num.	TDR cluster sizes	ADR cases (% of resistant)	loss cases (% of resistant)
14-03-16	18809	E	244 (1.3%)	164 (67.2%)	54 (22.1%)	82.75 (33.9%)	73.5	1-4	168.25 (69.0%)	7 (2.9%)
17-12-11	14035	E	189 (1.3%)	129 (68.3%)	43 (22.8%)	59.75 (31.6%)	54.5	1-4	133.25 (70.5%)	4 (2.1%)
17-12-06	5374	E	98 (1.8%)	76 (77.6%)	13 (13.3%)	20.50 (20.9%)	20.5	1-2	78.50 (80.1%)	1 (1.0%)
17-12-01	338	E	11 (3.3%)	9 (81.8%)		2.00 (18.2%)	2	1-2	9.00 (81.8%)	

Table S35. DRMs with prevalence > 0.5% found in position RT:K103 in C data set, and the evolution of their presence over time.

date	total samples	DRM	resistant cases (% of all)	resistant cases treatment-experienced (% of resistant)	resistant cases naive (% of resistant)	TDR cases (% of resistant)	TDR cluster num.	TDR cluster sizes	ADR cases (% of resistant)	loss cases (% of resistant)
14-03-16	18809	N	882 (4.7%)	605 (68.6%)	182 (20.6%)	317.12 (36.0%)	267	1-5	615.88 (69.8%)	51 (5.8%)
17-12-11	14035	N	654 (4.7%)	449 (68.7%)	139 (21.3%)	204.62 (31.3%)	184.5	1-3	472.38 (72.2%)	23 (3.5%)
17-12-06	5374	N	376 (7.0%)	286 (76.1%)	53 (14.1%)	83.00 (22.1%)	78	1-3	298.00 (79.3%)	5 (1.3%)
17-12-01	338	N	23 (6.8%)	18 (78.3%)	4 (17.4%)	4.50 (19.6%)	4.5	1-1	18.50 (80.4%)	

Table S36. DRMs with prevalence > 0.5% found in position RT:K219 in C data set, and the evolution of their presence over time.

date	total samples	DRM	resistant cases (% of all)	treatment-experienced (% of resistant)	naive (% of resistant)	TDR cases (% of resistant)	cluster num.	cluster sizes	ADR cases (% of resistant)	loss cases (% of resistant)
14-03-16	18809	E	109 (0.6%)	80 (73.4%)	15 (13.8%)	25.50 (23.4%)	24.5	1-3	83.50 (76.6%)	
17-12-11	14035	E	85 (0.6%)	65 (76.5%)	12 (14.1%)	17.00 (20.0%)	17	1-2	68.00 (80.0%)	
17-12-06	5374	E	54 (1.0%)	42 (77.8%)	6 (11.1%)	10.00 (18.5%)	10	1-2	44.00 (81.5%)	
17-12-01	338	E	5 (1.5%)	5 (100.0%)					5.00 (100.0%)	

Table S37. DRMs with prevalence > 0.5% found in position RT:K65 in C data set, and the evolution of their presence over time.

date	total samples	DRM	resistant cases (% of all)	treatment-experienced (% of resistant)	naive (% of resistant)	TDR cases (% of resistant)	cluster num.	cluster sizes	ADR cases (% of resistant)	loss cases (% of resistant)
14-03-16	18809	R	244 (1.3%)	199 (81.6%)	15 (6.1%)	38.50 (15.8%)	36.5	1-2	211.50 (86.7%)	6 (2.5%)
17-12-11	14035	R	177 (1.3%)	142 (80.2%)	11 (6.2%)	26.50 (15.0%)	25.5	1-2	153.50 (86.7%)	3 (1.7%)
17-12-06	5374	R	97 (1.8%)	79 (81.4%)	3 (3.1%)	13.00 (13.4%)	13	1-2	86.00 (88.7%)	2 (2.1%)
17-12-01	338	R	1 (0.3%)			0.50 (50.0%)	0.5	1-1	0.50 (50.0%)	

Table S38. DRMs with prevalence > 0.5% found in position RT:K70 in C data set, and the evolution of their presence over time.

date	total samples	DRM	resistant cases (% of all)	treatment-experienced (% of resistant)	naive (% of resistant)	TDR cases (% of resistant)	cluster num.	cluster sizes	ADR cases (% of resistant)	loss cases (% of resistant)
14-03-16	18809	R	196 (1.0%)	152 (77.6%)	19 (9.7%)	38.62 (19.7%)	35.5	1-4	161.38 (82.3%)	4 (2.0%)
17-12-11	14035	R	175 (1.2%)	140 (80.0%)	16 (9.1%)	28.62 (16.4%)	27	1-2	149.38 (85.4%)	3 (1.7%)
17-12-06	5374	R	123 (2.3%)	98 (79.7%)	10 (8.1%)	19.00 (15.4%)	19	1-2	105.00 (85.4%)	1 (0.8%)
17-12-01	338	R	14 (4.1%)	11 (78.6%)	1 (7.1%)	2.00 (14.3%)	2	1-1	12.00 (85.7%)	

Table S39. DRMs with prevalence > 0.5% found in position RT:M184 in C data set, and the evolution of their presence over time.

date	total samples	DRM	resistant cases (% of all)	treatment-experienced (% of resistant)	naive (% of resistant)	TDR cases (% of resistant)	cluster num.	cluster sizes	ADR cases (% of resistant)	loss cases (% of resistant)
14-03-16	18809	V	1009 (5.4%)	789 (78.2%)	79 (7.8%)	213.88 (21.2%)	197	1-4	833.12 (82.6%)	38 (3.8%)
17-12-11	14035	V	817 (5.8%)	642 (78.6%)	70 (8.6%)	154.38 (18.9%)	144.5	1-4	680.62 (83.3%)	18 (2.2%)
17-12-06	5374	V	524 (9.8%)	419 (80.0%)	41 (7.8%)	86.06 (16.4%)	80	1-4	443.94 (84.7%)	6 (1.1%)
17-12-01	338	V	53 (15.7%)	36 (67.9%)	3 (5.7%)	12.19 (23.0%)	9	1-4	41.81 (78.9%)	1 (1.9%)

Table S40. DRMs with prevalence > 0.5% found in position RT:M41 in C data set, and the evolution of their presence over time.

date	total samples	DRM	resistant cases (% of all)	treatment-experienced (% of resistant)	naive (% of resistant)	TDR cases (% of resistant)	cluster num.	cluster sizes	ADR cases (% of resistant)	loss cases (% of resistant)
14-03-16	18809	L	171 (0.9%)	117 (68.4%)	25 (14.6%)	51.72 (30.2%)	43.5	1-5	120.28 (70.3%)	1 (0.6%)
17-12-11	14035	L	146 (1.0%)	101 (69.2%)	20 (13.7%)	40.72 (27.9%)	33.5	1-5	106.28 (72.8%)	1 (0.7%)
17-12-06	5374	L	106 (2.0%)	79 (74.5%)	9 (8.5%)	22.28 (21.0%)	19	1-5	83.72 (79.0%)	
17-12-01	338	L	13 (3.8%)	10 (76.9%)		2.75 (21.2%)	2.5	1-2	10.25 (78.8%)	

Table S41. DRMs with prevalence > 0.5% found in position RT:S68 in C data set, and the evolution of their presence over time.

date	total samples	DRM	resistant cases (% of all)	treatment-experienced (% of resistant)	naive (% of resistant)	TDR cases (% of resistant)	cluster num.	cluster sizes	ADR cases (% of resistant)	loss cases (% of resistant)
14-03-16	18809	G	160 (0.9%)	51 (31.9%)	87 (54.4%)	103.25 (64.5%)	77	1-12	57.75 (36.1%)	1 (0.6%)
17-12-11	14035	G	113 (0.8%)	36 (31.9%)	59 (52.2%)	71.00 (62.8%)	56.5	1-10	43.00 (38.1%)	1 (0.9%)
17-12-06	5374	G	37 (0.7%)	16 (43.2%)	15 (40.5%)	19.00 (51.4%)	15.5	1-4	19.00 (51.4%)	1 (2.7%)
17-12-01	338	G	1 (0.3%)		1 (100.0%)	1.00 (100.0%)	1	1-1		

Table S42. DRMs with prevalence > 0.5% found in position RT:T215 in C data set, and the evolution of their presence over time.

date	total samples	DRM	resistant cases (% of all)	treatment-experienced (% of resistant)	naive (% of resistant)	TDR cases (% of resistant)	cluster num.	cluster sizes	ADR cases (% of resistant)	loss cases (% of resistant)
14-03-16	18809	Y	137 (0.7%)	97 (70.8%)	13 (9.5%)	37.97 (27.7%)	31.5	1-5	105.03 (76.7%)	6 (4.4%)
17-12-11	14035	Y	125 (0.9%)	89 (71.2%)	12 (9.6%)	32.97 (26.4%)	26	1-5	96.03 (76.8%)	4 (3.2%)
17-12-06	5374	Y	103 (1.9%)	76 (73.8%)	7 (6.8%)	20.78 (20.2%)	17.5	1-5	83.22 (80.8%)	1 (1.0%)
17-12-01	338	Y	17 (5.0%)	14 (82.4%)		2.75 (16.2%)	2.5	1-2	14.25 (83.8%)	

Table S43. DRMs with prevalence > 0.5% found in position RT:V106 in C data set, and the evolution of their presence over time.

date	total samples	DRM	resistant cases (% of all)	treatment-experienced (% of resistant)	naive (% of resistant)	TDR cases (% of resistant)	cluster num.	cluster sizes	ADR cases (% of resistant)	loss cases (% of resistant)
14-03-16	18809	M	381 (2.0%)	301 (79.0%)	36 (9.4%)	71.25 (18.7%)	66	1-4	319.75 (83.9%)	10 (2.6%)
17-12-11	14035	M	285 (2.0%)	225 (78.9%)	31 (10.9%)	47.25 (16.6%)	46	1-3	238.75 (83.8%)	1 (0.4%)
17-12-06	5374	M	151 (2.8%)	122 (80.8%)	11 (7.3%)	21.50 (14.2%)	21.5	1-2	130.50 (86.4%)	1 (0.7%)
17-12-01	338	M	8 (2.4%)	7 (87.5%)		1.50 (18.8%)	1.5	1-2	6.50 (81.2%)	

Table S44. DRMs with prevalence > 0.5% found in position RT:V108 in C data set, and the evolution of their presence over time.

date	total samples	DRM	resistant cases (% of all)	treatment-experienced (% of resistant)	treatment-naive (% of resistant)	TDR cases (% of resistant)	TDR cluster num.	TDR cluster sizes	ADR cases (% of resistant)	loss cases (% of resistant)
14-03-16	18809	I	194 (1.0%)	114 (58.8%)	55 (28.4%)	77.75 (40.1%)	72	1-3	123.25 (63.5%)	7 (3.6%)
17-12-11	14035	I	142 (1.0%)	83 (58.5%)	39 (27.5%)	53.25 (37.5%)	51	1-2	91.75 (64.6%)	3 (2.1%)
17-12-06	5374	I	70 (1.3%)	47 (67.1%)	13 (18.6%)	20.00 (28.6%)	19	1-2	51.00 (72.9%)	1 (1.4%)
17-12-01	338	I	3 (0.9%)	3 (100.0%)					3.00 (100.0%)	

Table S45. DRMs with prevalence > 0.5% found in position RT:V179 in C data set, and the evolution of their presence over time.

date	total samples	DRM	resistant cases (% of all)	treatment-experienced (% of resistant)	treatment-naive (% of resistant)	TDR cases (% of resistant)	TDR cluster num.	TDR cluster sizes	ADR cases (% of resistant)	loss cases (% of resistant)
14-03-16	18809	D	294 (1.6%)	99 (33.7%)	159 (54.1%)	214.50 (73.0%)	139	1-19	102.50 (34.9%)	23 (7.8%)
		E	120 (0.6%)	11 (9.2%)	34 (28.3%)	108.25 (90.2%)	24	1-80	12.75 (10.6%)	1 (0.8%)
17-12-11	14035	D	213 (1.5%)	71 (33.3%)	119 (55.9%)	146.50 (68.8%)	101	1-10	78.50 (36.9%)	12 (5.6%)
		E	37 (0.3%)	5 (13.5%)	19 (51.4%)	30.25 (81.8%)	15	1-15	6.75 (18.2%)	
17-12-06	5374	D	86 (1.6%)	32 (37.2%)	42 (48.8%)	50.75 (59.0%)	39.5	1-5	36.25 (42.2%)	1 (1.2%)
		E	8 (0.1%)	4 (50.0%)	3 (37.5%)	3.50 (43.8%)	3.5	1-1	4.50 (56.2%)	
17-12-01	338	D	10 (3.0%)	4 (40.0%)	3 (30.0%)	5.50 (55.0%)	5.5	1-2	4.50 (45.0%)	
		E								

Table S46. DRMs with prevalence > 0.5% found in position RT:Y181 in C data set, and the evolution of their presence over time.

date	total samples	DRM	resistant cases (% of all)	treatment-experienced (% of resistant)	treatment-naive (% of resistant)	TDR cases (% of resistant)	TDR cluster num.	TDR cluster sizes	ADR cases (% of resistant)	loss cases (% of resistant)
14-03-16	18809	C	419 (2.2%)	299 (71.4%)	56 (13.4%)	108.38 (25.9%)	98	1-4	321.62 (76.8%)	11 (2.6%)
17-12-11	14035	C	334 (2.4%)	234 (70.1%)	50 (15.0%)	80.62 (24.1%)	76.5	1-3	256.38 (76.8%)	3 (0.9%)
17-12-06	5374	C	183 (3.4%)	133 (72.7%)	21 (11.5%)	38.00 (20.8%)	36	1-2	146.00 (79.8%)	1 (0.5%)
17-12-01	338	C	17 (5.0%)	13 (76.5%)		2.00 (11.8%)	2	1-1	15.00 (88.2%)	
